# Trehalose May Decrease the Transmission of Zika Virus to the Fetus by Activating Degradative Autophagy

**DOI:** 10.3389/fcimb.2017.00402

**Published:** 2017-09-06

**Authors:** Shu Yuan, Zhong-Wei Zhang, Zi-Lin Li

**Affiliations:** ^1^College of Resources, Sichuan Agricultural University Chengdu, China; ^2^Department of Cardiovascular Surgery, Xijing Hospital, Medical University of the Air Force Xi'an, China

**Keywords:** autophagy, exosome, lysosomal fusion, trehalose, Zika virus

A widespread epidemic of Zika virus (a mosquito-borne flavivirus) infection was reported from 2015 in South and Central America. A major concern associated with the infection is the significantly increased incidence of microcephaly in fetuses born to the mothers infected with Zika virus (Mlakar et al., 2016). Researchers studying monkeys have shown that one infection with Zika virus protects the animal against future infections. Neutralizing antibodies are detected at 21 days post-infection. Re-challenge at 10 weeks after the initial inoculation resulted in no detectable viral replication, indicating successfully protective immunity against the virus (Dudley et al., 2016). They also found that non-pregnant animals could clear the virus within 10 days post-infection, however the virus persisted in the blood of pregnant monkeys for 35–70 days (Dudley et al., 2016). One possible explanation for the persistence of the virus in pregnancy is that the immune system of the mother was compromised, and she simply was not able to clear the virus as fast as the non-pregnant one. However, the pregnant animal (woman) still has a certain level of immunity. Both type I interferons and type III interferons are apparently induced by Zika virus infections, and the interferons have an ability to restrict Zika virus replication in human trophoblast cells (Bayer et al., 2016; Quicke et al., 2016). The other explanation, more provocative hypothesis is that the persistence of the virus is indicative of the fetal infection, and what they observed in the maternal serum was the shedding of virus by the fetus back into the mother's blood (Driggers et al., 2016). We cannot conclusively claim that the persistence of the virus does not reflect the fetal infection or there is no persistence found in non-pregnant animals (people), as it is possible that Zika virus may persist in immune-privilege cells (Hazlett and Hendricks, 2010). However, an analysis of the clinical data implies that the virus may take ~5 weeks to reach the fetus for most cases (Noronha et al., 2016; Soares de Souza et al., 2016; Yuan et al., 2017a). Therefore, the virus detected in the pregnant monkey within 35 days of infection is unlikely the backflow from the fetus back into the mother's bloodstream.

## Viral secretion in the exosome may be central to its immune evasion

**Figure 1:**
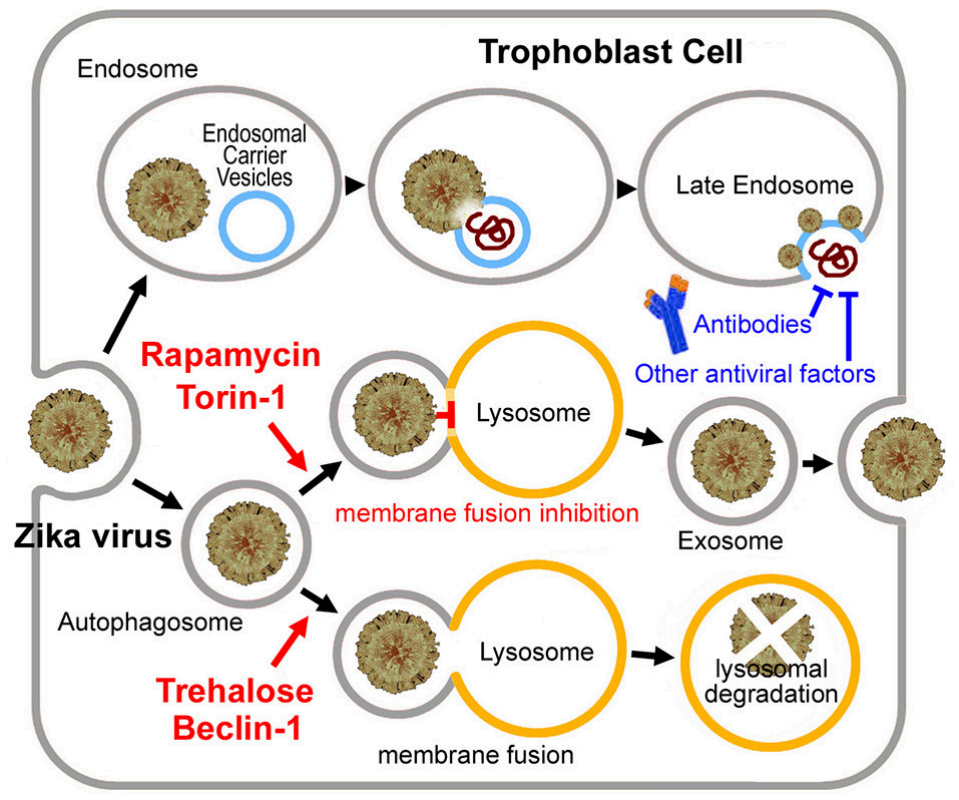
Putative Zika virus entry pathway and the exosome pathway in the trophoblast cell. For Zika virus, upon binding to the receptor, the virus enters the early endosome through the endocytic process. Several minutes later, the virus particle fuses with the endosomal carrier vesicle (ECV) membrane predominantly. However, for a short time, viral nucleocapsids remain trapped in the ECV lumen, until ECV fuses back with the late endosome membrane. Then the nucleocapsid is released. Alternatively, the virus may enter the autophagosome, and then to be degradated by fusing with the lysosome or become a sort of cargo of the placental exosome by blocking the autophagosome-lysosome fusion. Exosome may provide the virus with a shield from the mother's immune system. Neutralizing antibodies or other antiviral factors cannot work on the virus embedded in the exosome. Torin 1 or rapamycin induces exosome aggregation with the virus embedded in, if the lysosomal fusion step was blocked. While Beclin 1 or trehalose rescues the impaired fusion step, which results in lysosomal degradation of the virus.

Then why was the long-time persistence of the virus only observed in pregnancy? We previously hypothesized that Zika virus may hide in the exosome, which forms a shield against the mother's immune system (Zhang et al., 2017). There is evidenced that Hepatitis C virus, another flavivirus, can be transmitted through exosomes and utilize the autophagy pathway for viral transmission thus evading antibody-mediated immune responses (Ramakrishnaiah et al., 2013; Longatti, 2015; Shrivastava et al., 2015). Like Hepatitis C virus, Zika virus may infect trophoblast cells by entering the endoplasmic reticulum of the trophoblast to become a sort of cargo of the placental exosome (Adibi et al., 2016; Zhang et al., 2017), which is closely linked with the “secretory autophagy” process. In contrast to degradative autophagy (fusion with the lysosome), the secretory autophagy may result in secretion or expulsion of viral particles instead of their degradation (Figure 1) (Chahar et al., 2015; Ponpuak et al., 2015; Carneiro and Travassos, 2016).

## The roles of autophagy in flavivirus entry and replication

The roles of autophagy in flavivirus entry and replication have been well-studied. In general, the virus customizes autophagy proteins for efficient viral entry (Li et al., 2012; Dong and Levine, 2013; Jackson, 2015). For Hepatitis C virus, autophagy appears be required for initiation of, but not maintenance of, viral replication (Dreux et al., 2009). For Dengue virus, viral entry, replication, and translation have all been linked to the autophagic pathways (Panyasrivanit et al., 2009). Zika virus infection is accompanied with the observation of a lot of double membrane autophagosomes (Hamel et al., 2015; Souza et al., 2016; Lennemann and Coyne, 2017). While the co-detection of the virus envelope protein and the autophagy marker protein LC3 (cytosolic microtubule-associated light chain 3) has been reported (Hamel et al., 2015). Thus, some autophagosome formation inhibitors (such as 3-methyladenine and wortmannin) strongly reduced viral copy numbers in some cell lines (Nour et al., 2013; Hamel et al., 2015). However, some opposite reports suggest that the roles of autophagy in flavivirus infections are controversial (Li et al., 2016; Rolfe et al., 2016). ATG16L2 (Autophagy related 16-like 2) was identified among the top 30 down-regulated genes in human neural stem cells after the Zika virus infection (Rolfe et al., 2016). *LC3* transcript was also repressed by the Zika virus infection in mouse brain cells (Li et al., 2016). However, in these two opposite reports, the gene expression analyses were performed at 56–72 h after the virus inoculation, which are late infection stages. Declines of some autophagy-related genes at the late infection stages do not mean that autophagy was inhibited at the early infection stages or at the entry steps.

Depletion of autophagy-related (ATG) protein ATG5 does not affect replication of West Nile virus in some cell lines (Vandergaast and Fredericksen, 2012). Some other reports even indicated that flavivirus replication levels were increased in autophagy-deficient cells. For example, West Nile virus replication was increased in mouse embryonic fibroblast cells depleted of ATG5 (Kobayashi et al., 2014). For Japanese Encephalitis virus, either depletion in ATG7 or deficiency in ATG5 would result in higher viral replication levels in mouse embryonic fibroblast cells (Sharma et al., 2014). However, these results do not mean that autophagy down-regulates viral replication directly. Autophagy may play a positive role in the early infection stages; however it becomes dysfunctional when the misfolded proteins accumulate at the late stages. Autophagy-deficient cells may be highly susceptible to virus-induced cell death (Sharma et al., 2014; Martín-Acebes et al., 2015). Therefore, higher viral loads were detected in these susceptible cells.

## Flavivirus infection inhibits autophagosome-lysosome fusion

In the case of Dengue virus, early after the infection, basal, and activated autophagic fluxes were enhanced. However, during the established viral replication, basal, and Torin 1-activated autophagic fluxes were declined because of a block to autophagic vesicle formation and reduced autophagic degradation capacity (Metz et al., 2015). During the late stages of Dengue virus infection, autophagic vesicles increased as a result of inefficient fusion of autophagosomes with lysosomes, although the lysosomal activities were increased (Metz et al., 2015). Similar autophagosome-lysosome fusion defect may also occur in the Zika virus infection, since that a lot of genes required for the lysosomal fusion were down-regulated by the Zika virus infection (Li et al., 2016), such as *RAB7* (a member of the Rab GTPase superfamily) (Pankiv et al., 2010) and genes of class C Vacuolar protein sorting (Vps)/HO motypic fusion and Protein Sorting (HOPS) tethering complex (*Vps16, Vps18, Vps33*) (Wurmser et al., 2000). If the lysosomal fusion step was blocked, up-regulating autophagosome formation may cause exosome aggregation with the virus embedded in Zhang et al. (2017). This assumption is supported by the fact that Torin 1 (a classic mechanistic target of rapamycin mTOR-dependent autophagy activator) greatly enhanced Zika virus replication (Hamel et al., 2015), and rapamycin treatment significantly enhanced Dengue virus replication (Lee et al., 2013; Chu et al., 2014). Because of the impaired autophagosome-lysosome fusion, the Zika virus may become a cargo of the placental exosome, other than to be degradated in the lysosome, which may be central to its immune evasion as observed in the pregnant animals (Figure 1).

## Trehalose may be an idea drug with a high safety

Trehalose induces autophagosome formation in an mTOR-independent pathway and rescues the impaired lysosomal fusion (Ejlerskov et al., 2013). The roles of trehalose on autophagy in some other diseases have been reported before. For example, the defect in autophagosome-lysosome fusion has been observed in the amyotrophic lateral sclerosis (ALS) model mice of motor neuron degeneration. Interestingly, like flavivirus infections, rapamycin showed adverse effects to the ALS disease progression (Zhang et al., 2011). On the contrary, trehalose rescued the impaired fusion step, which resulted in aggregated autophagic degradation in the motor neurons. Trehalose is able to attenuate the autophagic flux defect and improve ALS disease course (Zhang et al., 2014; Yuan et al., 2017b). Besides ALS, a similar block to the autophagy-lysosome degradative pathway has been also reported in the mouse model of human tauopathy. Stimulation of autophagy by trehalose reduced tau aggregates and improved neuronal survival in the cerebral cortex and the brainstem (Schaeffer et al., 2012). Trehalose also enhances the degradative capacity of macrophages and is considered as a therapy for atherosclerotic vascular disease (Sergin et al., 2017). Therapeutic effects of trehalose on virus infections have been proved recently that trehalose had a profound inhibitory effect on Human cytomegalovirus replication and strongly inhibited viral spread through activating degradative autophagy presumably (Belzile et al., 2015).

As discussed in our previous analysis (Yuan et al., 2017a), the placental transfer of Zika virus may be a time-consuming process. The virus may take about 5 weeks to reach the fetus (or over 12 weeks, if the infection occurs early in pregnancy). People could postulate that if most Zika virus was cleared before it reaches the fetus, the incidence of microcephaly may be largely decreased. Trehalose promotes the autophagosome-lysosome fusion and prevents the virus from entering the exosome, and therefore induces viral degradation or makes the virus exposed to the mother's immune system (Figure 1). Hence the trehalose treatment might help to clear the virus within 5 weeks. Trehalose therefore may be useful for early Zika virus infections. However, trehalose may not prevent Zika virus induced microcephaly in the late infection stages when the virus has been already reached the fetus. Considering that the mother's immune system would spend more than 10 days to clear the virus, trehalose treatment should be applied as soon as possible after the infection.

During pregnancy, when the number of candidate drugs is exceedingly limited and the bar for the clinical approval is extremely high, people must be very cautious when testing any potential therapies that could be used in human pregnancy. Trehalose is non-reducing disaccharide, with stable chemical property and multiple protective effects to organisms and biological macromolecules (Richards et al., 2002). It is a kind of food, but not a drug, and does not produce any significant side effects. Trehalose at effective intracellular concentrations does not impair of mouse or rat fetus development or show any teratogenic effect (Richards et al., 2002; Eroglu et al., 2003). However, clinical trials to assess its embryotoxicity or teratogenicity to humans are still lacking. Therefore, only after careful toxicological tests in humans, trehalose treatment could be used as a promising therapy for the pregnant women infected with Zika virus.

## Future clinical applications

The optimal dosage of trehalose for humans needs clinical investigations. In the mouse experiments, 2% trehalose containing water was given to the mouse through *ad-libitum* consumption, and these oral administrations showed significant therapeutic effects (Schaeffer et al., 2012; Zhang et al., 2014). Whether similar trehalose treatments should be given for humans needs further studies. There is no recommendation from the FDA on this sugar. It would be well to follow the WHO guideline and restrict intake of all sugars to 50 g *per* day (46). The normal person daily potable water quantity is about 1,500–2,500 ml. Two percentages trehalose means 30–50 g *per* day. Thus, the WHO guideline may be a feasible one. However, pregnant women also intake other sugars from their diets. High maternal intake of free sugars during pregnancy is associated with increased risks of many diseases in the offspring, such as atopic asthma and allergic asthma (Bédard et al., 2017; Torjesen, 2017). Appropriate oral dose needs further studies.

Nevertheless, a large part of trehalose would be broken down into glucose by the trehalase on the intestinal mucosa (Richards et al., 2002), oral administration may not be a very effective method. Trehalose injection might be an alternative option (Echigo et al., 2012; Sergin et al., 2017), although the tests in the rhesus macaque model should be performed before the advancement to human clinical trials.

## Author contributions

SY coordinated the writing and wrote this manuscript together with inputs form all other listed co-authors. All authors made equal contributions in finalizing this manuscript.

### Conflict of interest statement

The authors declare that the research was conducted in the absence of any commercial or financial relationships that could be construed as a potential conflict of interest. The reviewer SR and handling Editor declared their shared affiliation, and the handling Editor states that the process nevertheless met the standards of a fair and objective review.
